# Supplementary material for: The interactome of intact mitochondria by cross-linking mass spectrometry provides evidence for coexisting respiratory supercomplexes
Source: Mol Cell Proteomics. 2017 Dec 8;17(2):216–32. doi: 10.1074/mcp.RA117.000470 (PMC5795388; doi:10.1074/mcp.RA117.000470)
Supplement: Supplemental Data [file supp_RA117.000470_133922_0_supp_23978_fzffwf.zip › spectra_annotation/mito_spectra_annotation/239-1-3-1-14-1.pdf]

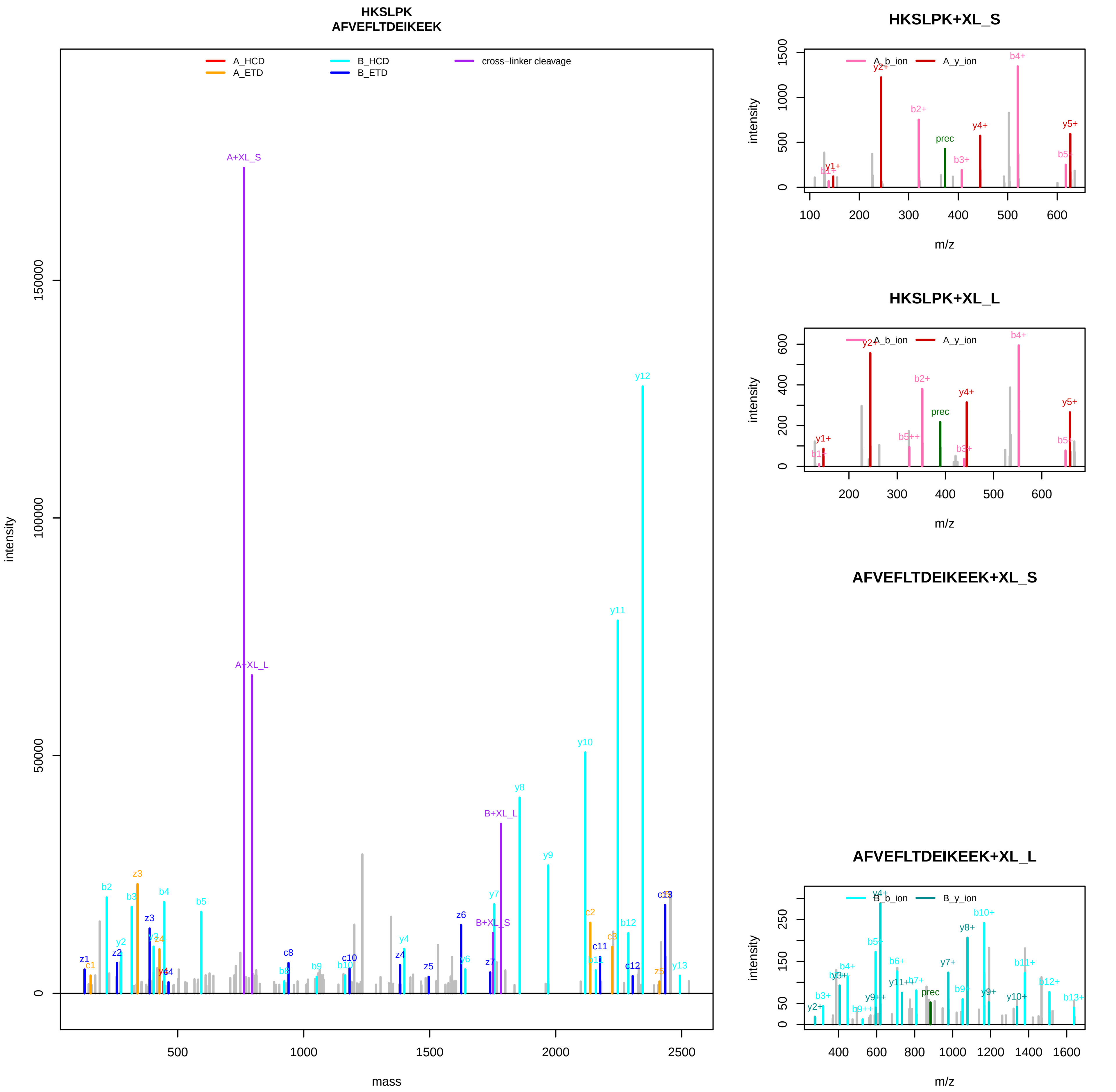
HKSLPK
AFVEFLTDEIKEEK
A_HCD
A_ETD
B_HCD
B_ETD
cross−linker cleavage
A+XL_S
A+XL_L
B+XL_L
B+XL_S
y12
y11
y10
y8
y9
y7
c13
c2
c3
b12
c11
c12
y13
z6
z7
y6
z5
z4
y4
c10
b10
b9
c8
b8
b5
b4
b3
b2
z3
z2
z1
c1
y2
c4
intensity
mass
0
50000
100000
150000
500
1000
1500
2000
2500
HKSLPK+XL_S
A_b_ion
A_y_ion
y2+
b2+
prec
b3+
y4+
b4+
b5+
y5+
y1+
b1+
m/z
intensity
0
500
1000
1500
100
200
300
400
500
600
HKSLPK+XL_L
A_b_ion
A_y_ion
y2+
b2+
b5++
prec
y4+
b3+
b4+
y5+
b5+
y1+
b1+
m/z
intensity
0
200
400
600
200
300
400
500
600
AFVEFLTDEIKEEK+XL_S
AFVEFLTDEIKEEK+XL_L
B_b_ion
B_y_ion
y4+
b10+
y8+
b6+
b5+
y7+
b11+
b12+
b13+
y10+
y9+
b9+
prec
b7+
y11++
y9++
b9++
b4+
y3+
b3+
y2+
m/z
intensity
0
50
150
250
400
600
800
1000
1200
1400
1600
